# Scepticaemia: The impact on the health system and patients of delaying new treatments with uncertain evidence; a case study of the sepsis bundle

**DOI:** 10.12688/f1000research.14619.2

**Published:** 2018-07-05

**Authors:** Robin Blythe, David Cook, Nicholas Graves

**Affiliations:** 1 Australian Centre for Health Services Innovation, School of Public Health and Social Work, Institute for Health and Biomedical Innovation, Queensland University of Technology, Brisbane, Queensland, Australia; 2 Intensive Care Unit, Princess Alexandra Hospital, Brisbane, Queensland, Australia

**Keywords:** Sepsis, Costs and Cost Analysis, Evidence-based practice

## Abstract

Background: A sepsis care bundle of intravenous vitamin C, thiamine, and hydrocortisone was reported to improve treatment outcomes. The data to support it are uncertain and decision makers are likely to be cautious about adopting it. The objective of this study was to model the opportunity costs in dollars and lives of waiting for better information before adopting the bundle.

Methods: A decision tree was built using information from the literature. We modelled the impact of bundle adoption under three scenarios using a simulation in which the bundle was effective as reported in the primary trial, less effective based on other information, and ineffective.

The measurements were health services costs, quality-adjusted life years, and transition probabilities.

Results: If the bundle proves to be effective under either scenario, it will save billions of dollars and millions of life-years in the United States. This is the opportunity cost of delaying an adoption decision and waiting for better quality evidence. We suggest that hospital decision-makers consider implementing the bundle on a trial basis while monitoring costs and outcomes data even while the evidence base is uncertain.

Conclusions: If the decision maker is unwilling to use the best available evidence now, but rather wishes to wait for definitive evidence they are risking incurring large costs for health care systems and for the patients they serve. An explicit analysis of uncertain clinical outcomes is a useful adjunct to guide decision making where there is clinical ambiguity. This approach offers a valid alternative to the default of clinical inactivity when faced with uncertainty.

## Introduction

Sepsis arises frequently among patients admitted to hospitals in the US and elsewhere. It is often fatal, accounting for 30% to 50% of inpatient deaths ^1^. Those who survive incur large costs from increased risk of organ damage ^2^. A recent paper by Marik *et al.* (2017) described the effectiveness of a treatment bundle made up of intravenous vitamin C, thiamine and hydrocortisone ^3^. Because of small sample size, non-randomised design, large observed treatment effects and the simple and low cost characteristics of the bundled intervention, there was scepticism among clinicians, administrators and payers regarding adoption ^4– 6^. Rapid adoption without good evidence has risks and costs, yet delaying an effective treatment imposes larger costs to patients and the health system. Waiting for conclusive evidence might be a poor and costly strategy and should be balanced against the likely costs of rapid adoption with uncertain and low quality evidence.

The aim of this paper is to estimate the economic consequences of a decision to adopt the Marik sepsis bundle early, under conditions of large uncertainty. This is compared to the alternative, which is to wait 2.5 years; the time period between publication of the first paper in June 2017 ^3^ and the completion of several multi-centre trials of the bundle in late 2019. The trials will ideally reduce the uncertainty in an adoption decision. This paper predicts the cost savings and health benefits of adopting the Marik bundle immediately with uncertain evidence for the entire United States health system, compared to waiting for better quality evidence. Our estimates examine likely changes to costs and health outcomes if the treatment is found to be effective, less effective and ineffective.

## Methods

**Figure 1.**
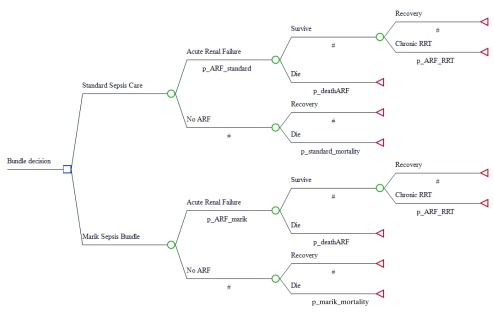
Sepsis decision tree model using TreeAge Pro 2017.

An incremental cost-effectiveness analysis was conducted on the change to costs and Quality-Adjusted Life Years (QALYs) of standard sepsis care compared to the early adoption of the Marik bundle ^3^. Values for expected costs and health utilities were taken from the literature, with probabilities of treatment outcomes taken from Marik’s paper ^3^. The time horizon was 5 years in order to sufficiently measure the long-term outcomes of acute renal failure (ARF), an important and common result of severe septic shock ^2^. A decision tree was programmed in TreeAge Pro 2017 R2.1 (Williamstown, MA2017) and prior statistical distributions of costs, outcomes and probabilities used to include uncertainties in the data, see Figure 1. Patients receiving either modality progress through a chance node to ARF or not. Patients progress through a second chance node, where they either recover or die. Surviving ARF patients progress through a final chance node where they may require chronic renal replacement therapy (RRT), which can take the form of either dialysis or organ transplantation ^2^.

### Costs

The costs of care include the hospital cost of a sepsis episode, the additional cost of the Marik treatment bundle, the additional cost of an episode of ARF, and the annual ongoing cost of RRT. Hospital costs were taken from a systematic review by Arefian *et al.* (2017) for a US perspective with a mean of $32,421 and standard deviation of $15,051 per stay ^7^. For the Marik bundle, we used an estimate of $528 and a standard deviation of 5% at $26.50 per patient per course. The additional cost of a hospital episode of ARF was taken from Silver *et al.* (2017) as $11,016 with a standard error of $279.59 ^8, 9^. The annual cost of RRT for end stage renal disease varies depending on treatment modality, such as dialysis or transplant, and a weighted average of $76,936, with a standard deviation of $15,387 was taken from the 2017 USRDS Annual Data Report ^10^.

### Health utilities

The health utility score of patients reflects the value of their heath state and is a measure of health related quality of life. It is expressed on a range between zero, the worst health state, and one, the best possible health state ^11^. The scores for patients who recover from septic shock depend on whether or not they suffered ARF. Patients who did not suffer ARF still underwent the stress of intensive care unit recovery, and their mean health utility scores and standard deviations were taken from Cuthbertson *et al.* (2010) at 1, 2.5, and 5 years as 0.666 (0.280), 0.701 (0.281), and 0.677 (0.301), respectively ^12^. Survivors of acute kidney problems were found to have a utility of 0.40 with a standard deviation of 0.37 at 60 days. We did not find evidence of improved health utilities over the 5 year period ^13^. The health utility of death was valued at 0 ^11^.

### Transition probabilities

The transition probabilities of patients moving through the decision tree were informed by data from Marik’s study. This includes the probabilities of survival and ARF, see Table 1 ^3^. In Marik’s study, the Marik bundle’s probability of death and ARF were 0.085 and 0.097, with standard errors of 0.041 and 0.043 respectively. These compared to the standard care mortality and ARF probabilities of 0.404 and 0.234, with standard errors of 0.071 and 0.062 respectively. Full transition probabilities are available in Dataset 1.

**Table 1.** Transition probabilities from bundle decision to survival and recovery.

	Mean Standard Care (SE)	Mean Standard Care, Scenario 1 (SE)	Mean Marik Bundle (SE)
Mortality	0.404 (0.071)	0.255 (0.064)	0.085 (0.041)
ARF	0.234 (0.062)	0.170 (0.055)	0.097 (0.043)

### Modelling additional scenarios

Scenario 1 uses probabilities from the literature for mortality and ARF; costs and utilities were unchanged. The attributable mortality risk and rate of ARF in the general population vary in the literature and are different to those reported in Marik’s control cohort. We used the mortality risk associated with ARF identified in Oppert *et al.* (2008) of 21.2% and the rate of sepsis-associated ARF in Lopes *et al*. (2009) of 17.0% ^14– 17^. These are lower than the rates seen in Marik’s observation group. This led to an overall mortality rate of 25.5%, consistent with several studies on septic shock ^15, 18^.

Scenario 2 represents a worst-case scenario, in which Marik’s bundle is ineffective and there is no difference in probabilities between the bundle and standard care. We modelled scenario 2 using the same transition probabilities found in scenario 1 for both arms of the decision tree. As in scenario 1, costs and utilities remained unchanged, though we carried the worst-case QALY outcome to illustrate the unlikely possibility of minor harm.

### Probabilistic sensitivity analysis

Probabilistic sensitivity analysis was conducted on the decision tree by taking 1000 random resamples of values from the prior statistical distributions of model parameters. This propagates all uncertainty in the data forward to the results and provides useful information for decision making.

## Results

The total economic cost of patients under standard care, including 5 year estimations of RRT, was $41,982 compared to the total economic cost of patients receiving the Marik bundle of $35,867, an expected saving of $6,115 per patient. The annual incidence of severe sepsis in the United States is around 300 cases per 100,000 population, suggesting a total expected cost saving over 2.5 years of $14.9bn USD ^9^. Over the same period, patients gain an additional 1.46 QALY per case, or 3.6m QALYs over 2.5 years. Given the reduced costs and improved outcomes, the Marik bundle dominates standard care, as it both saves costs and increase health outcomes at the same time. The probabilistic sensitivity analysis shows this conclusion arises 93.6% of the time.

Scenario 1 reduces the mean expected economic cost of standard care to $38,068 per patient, compared to the Marik bundle at $35,478. The expected cost saving between the bundle and standard care was $2,590 per patient, a total saving of $6.3bn USD. Altering the transition probabilities to values from the literature also reduces the QALYs gained, with patients gaining 0.46 QALY per case, or 1.1m QALY over 2.5 years. In this scenario, the Marik bundle still dominates standard care by saving costs and improving health outcomes. The probabilistic sensitivity analysis shows this conclusion arises 87.8% of the time.

Scenario 2 is that there is no change to outcomes between Marik’s bundle and standard care. The mean cost per patient over 5 years is $37,022USD for standard care and $37,550USD for the Marik bundle, an increase of $528 per patient over a 5 year period. There is no difference in QALYs between treatment alternatives. We were unable to find any evidence of harm to the patient as a result of the bundle. Hydrocortisone and thiamine are already present in routine sepsis care, and the dosage of 6g of vitamin C per day has been shown to be safe unless contraindicated ^19– 21^. In this scenario, assuming the intervention is universally adopted, no patients will have been harmed, but healthcare payers and providers would have spent an additional $1.3bn USD over 2.5 years. For the sake of illustration we have carried the worst possible health outcome from scenario 1 to scenario 2. The probabilistic sensitivity analysis shows a negative health outcome arises 2.5% of the time. Outcomes from the scenario analyses are listed in Table 2 below.

**Table 2.** Scenario analyses for adoption compared to baseline adoption outcomes.

	Cost savings	95% uncertainty interval	QALYs gained	95% uncertainty interval
Baseline	$14.9bn	[$-7.3bn – $33.9bn]	3.6m	[1.3m – 6.3m]
Scenario 1: less effective	$6.3bn	[-$8.4bn – $18.3bn]	1.1m	[-0.1m – 2.6m]
Scenario 2: ineffective	-$1.3bn	[-$0.9bn – -$0.7bn]	0	[-0.1m – 0]

Sepsis Distributions Table Data on distributions used in the analysis. Click here for additional data file. Copyright: © 2018 Blythe R et al. 2018 Data associated with the article are available under the terms of the Creative Commons Zero "No rights reserved" data waiver (CC0 1.0 Public domain dedication).

## Discussion

We found that adopting the Marik bundle has a high likelihood to save billions of dollars and generate millions of extra QALYs under the conditions outlined in Marik’s paper and in an alternate scenario that uses other data. Under the ineffective treatment of scenario 2 costs are increased to health services by $0.5bn per year. These results reveal substantial opportunity costs in dollars and lives if we fail to implement and the bundle is ultimately found to be effective, even if the treatment effect is lower than purported by Dr Marik. If the bundle does not work then some costs have been incurred by hospitals for no heath gain. Not adopting the bundle because the evidence for effectiveness is currently uncertain could well be a poor strategy. The worst possible outcome from the base case is that we spend around $5,700 per QALY; given a conventional $50,000/QALY valuation, this gives us a net monetary benefit of $108bn over 2.5 years ^22^.

The scenario analysis uses values at which the Marik bundle is less effective, specifically by aligning observation group figures with the literature. The mortality rate of sepsis under standard care was suggested by Marik to be 40.4%. Sepsis mortality has been declining in the US, from 46.9% in the early 1990s to 21.2% in 2014, declining by about 3% per annum and driven by improved organ support systems and protocoled early recognition and treatment ^17, 23, 24^. It might be that Marik’s study featured unusually sick patients in the control group. Study participants are often unrepresentative of the general population and the intervention group may have been less likely to suffer an adverse outcome ^25^. This progress in sepsis care management is complicated by the fact that claims data has shown concurrently increasing sepsis incidence and decreasing mortality, so the literature is conflicted ^26, 27^. It is possible that the increased incidence from claims data is due to increased reimbursement received by US hospitals for sepsis compared to other diseases, and patients are being misdiagnosed. An increasing rate of misdiagnosis of patients that do not have sepsis increases the denominator of septic cases while mortality stays the same in the numerator, creating the illusion of declining mortality ^17, 27^.

Regarding the mortality of Marik’s treatment group, we note the significant improvement in outcomes recommending the use of steroids in adults with septic shock by Annane *et al.* (2018) ^28^. Patients randomised to the steroid group (n = 614) showed a 6% absolute reduction in 90-day all-cause mortality when compared to placebo (n = 627). Therefore, the figures in Marik’s patient cohort may have been unusual, but they are plausible.

There is no current body of evidence that suggests this bundle is dangerous. Indeed, the combination of vitamin C and thiamine with steroids would have to cause an attributable death once in every 10 sepsis patients - by a hitherto unimagined and novel mechanism - to negate the modelled benefits of its early adoption.

### Limitations

Our study excluded the costs of bundle implementation, including training, labour and the potential for high costs of de-implementing an unsuccessful treatment. These would have increased the cost of bundle implementation, so the incremental cost of the Marik bundle may be understated. We noted that hydrocortisone, as part of the bundle, was found by Venkatesh *et al.* (2018) to speed up resolution of shock and reduce the need for blood transfusions, so the in-hospital cost-savings for the treatment group may also be understated ^21^. We were also unable to quantify the potential QALY gains from reductions in post-sepsis syndrome associated with the Marik bundle, understating the gains in utility from the bundle. There is also considerable uncertainty around the parameter estimates that are available. We did not have access to primary data, including clinical data on costs and transition probabilities for each patient, and were reliant on the literature.

### Bundle adoption decision

An average hospital in the US may treat around 230 sepsis patients per year ^16^. By implementing the bundle, it will spend an additional $528 per patient, or $121,440 per year. Conservative estimates from the scenario analysis shows cost savings of $2,590 and a gain of 0.46 QALYs per patient. If the treatment was effective for 47 patients out of 230, or 1 in 5, it will have paid for itself in terms of total economic costs. Comparing implementing the sepsis bundle to other hospital-based treatment studies shows that for 230 patients, the bundle costs less than a tenth of a standard phase I clinical trial, which run from $1.4m-$6.5m ^29^.

The case for not adopting the Marik bundle has several components. Scientific and empirical evidence is thin, and a single-site Vitamin C trial showing remarkable results only to be proven ineffective after a multi-site RCT is a clinical trope. If the bundle was ineffective, health systems will have added an unnecessary load to clinicians and implementers, which would then have to be de-implemented. Administrators and clinicians may be less likely to adopt novel treatments in the future. Introducing the bundle on the current evidence may also set a bad precedent for novel treatments, eroding the authority of the presiding physician and giving more credence to largely unproven interventions. The proposal in this paper does not replace the need for the clarity provided by good science and empirical research, but these are not always immediately available. We provide an approach to explicitly guide the interim decisions that must be made under these circumstances.

The Marik bundle is a somewhat unusual case relative to most ‘miracle’ interventions later found to be ineffective. As the analysis shows, it is an extremely cheap treatment with the potential to reduce rates of mortality and kidney damage at no risk to the patient. The delay between publication of the pilot study and results of the large RCT due in 2019 could create substantial opportunity costs in dollars and lives, and while there is not perfect evidence, under the circumstances it might be sufficient for hospitals and health systems to choose whether to conduct their own trials and not only independently verify their results, but also publish their findings and improve the availability of evidence around the treatment bundle.

The implementation decision of the Marik bundle relies upon the willingness of health administrators to use the available evidence to influence policy. We attempted to make the bundle choice as intuitive as possible, using straightforward trade-offs, simple modelling techniques, and a realistic decision process. Merlo *et al.* (2014) showed that for research to be accessible to decision-makers, it must be contextually relevant, contain little jargon, and put the terms of the implementation decision into terms that specify a trade-off that will be familiar to decision-makers ^30^. If the decision maker is unwilling to use the best available evidence now, but rather wishes to wait for definitive evidence they are risking incurring large costs for health care systems and for the patients they serve.

## Conclusion

An explicit analysis of uncertain clinical outcomes is a useful adjunct to guide decision making where there is clinical ambiguity. This approach offers a valid alternative to the default of clinical inactivity when faced with uncertainty.

## Data availability

The data referenced by this article are under copyright with the following copyright statement: Copyright: © 2018 Blythe R et al.

Data associated with the article are available under the terms of the Creative Commons Zero "No rights reserved" data waiver (CC0 1.0 Public domain dedication). 

Dataset 1. Sepsis Distributions Table: Data on distributions used in the analysis. 10.5256/f1000research.14619.d209630 ^31^
