# Supplementary material for: Scepticaemia: The impact on the health system and patients of delaying new treatments with uncertain evidence; a case study of the sepsis bundle
Source: F1000Res. 2018 Jul 5;7:500. Originally published 2018 Apr 26. [Version 2] doi: 10.12688/f1000research.14619.2 (PMC5989143; doi:10.12688/f1000research.14619.2)
Supplement: Peer review history

# Peer review: Referee response for version 1

**DOI:** 10.5256/f1000research.15909.r34658

**Published:** 2018-06-04

**Authors:** Adam Visser

**Affiliations:** 1 Toowoomba Hospital, Toowoomba, Qld, Australia

This fascinating paper uses a simple health economics model to argue that delaying the widespread implementation of the Vitamin C protocol as described by Marik and colleagues would lead to the loss of enormous potential savings to the health industry.  The argument is well presented, easily followed, and sound, with some exceptions.

 There is likely to be some inaccuracy in the figures presented.  The authors state that there are 1.5 million cases of sepsis in the United States yearly (no reference supplied, but data easily available via the CDC).  Whilst there may indeed be this number who meet the recently updated definition of sepsis, this large cohort of patients are different to those who have a mortality rate of 40% with standard care.  This group, as described in Marik’s paper, are a much sicker population.  Their number is likely to be significantly fewer than 1.5 million.  Whilst it is not known whether Vitamin C may have benefits in a less sick population, it is unlikely that ‘early adopters’ of this therapy will consider it in those that are not towards the severe end of the sepsis spectrum.  Reducing the exposed population in the model from 1.5 million will reduce the proposed savings by a proportional amount.

 Similarly, the quoted incidence of requirement for renal replacement therapy is overstated.  It is true that the Marik Vitamin C-treated group had an incidence of RRT-requiring AKI of 9.7%.  However, the Marik paper does not state that these patients all went on to require ongoing RRT for ESRF (at the authors’ quoted cost of $77,000 per year).  The majority of patients who require RRT in the setting of sepsis-induced AKI will recover their renal function either completely, or enough that they do not require ongoing dialysis.  Again, reducing the stated financial benefits of this aspect of the Vitamin C treatment will reduce the long-term economic attractiveness of the therapy.

 Overall however, the authors are correct in their statement that the economic implications of adopting the ‘bundle’ will be positive.  Furthermore, the economic implications should be secondary to the beneficial effects the treatment may have on mortality.  Again, this therapy is unlikely to be offered to 1.5 million patients per year, but whatever the number, those who receive it are likely to benefit. 

 A major strength of the paper is their use of a more pessimistic scenario (scenario 1) than published in the Marik study to demonstrate the robustness of their figures.  The Marik-stated improvement in mortality, from 40% to 8%, is likely to not be repeated in larger studies, as it is simply too great to be plausible.   Reducing the relative risk reduction such that mortality is reduced from 21% to 8% may be more realistic, and yet the economic figures still add up to considerable savings.

 It would perhaps also be useful to see a model where baseline mortality of 40% (ie the very sick cohort from Marik) have their mortality reduced to, say, 30% (ie a more plausible magnitude of effect from Vitamin C). 

 The authors have done well to pick this particular therapy to demonstrate their model.  The Vitamin C bundle is unique in recent years, in that it is a cheap and seemingly safe therapy.  Their model is unlikely to be able to be used for supporting other therapies that emerge in coming years, given the likelihood that these therapies will come from the pharmaceutical industry and have large price tags attached.

I have read this submission. I believe that I have an appropriate level of expertise to confirm that it is of an acceptable scientific standard.

# Peer review: Referee response for version 1

**DOI:** 10.5256/f1000research.15909.r33910

**Published:** 2018-05-11

**Authors:** Paul E. Marik

**Affiliations:** 1 Division of Pulmonary and Critical Care Medicine, Eastern Virginia Medical School, Norfolk, VA, USA

This is an outstanding paper that objectively evaluates the potential benefit of the vitamin C protocol for sepsis.This evaluation was impeccably conducted and reported. The impact of this paper is significant.

 A few comments that will improve the paper:

 1.The authors state " *The delay between publication of the pilot study and results of the large RCT due in mid-2018 could create substantial opportunity costs"*. This study which the authors cite is the NIH funded study due for completion in mid-2018 which is investigating vitamin c (ALONE) and not the combination AND in patients with ARDS (not specifically sepsis). Secondly, this study is not powered for a mortality difference. Currently according to Clintrials.gov there are 8 RCTS testing the combination and 3 testing Vitamin C alone. This suggests that it may take at least until the end of 2019 before the results of any of these studies are available and we can make firm conclusions.

 2. The authors refer to the "sepsis bundle"; I would avoid using this term as it will create confusion with the Surviving Sepsis Bundle.. widely known as the Sepsis Bundle. I would call it the Vitamin C protocol, the Marik Protocol, or something similar.

 3. Approximately 50% of sepsis survivors develop the post-sepsis syndrome, defined as severe physical and cognitive dysfunction not unlike PTSD.  This syndrome is associated with enormous suffering and high costs. It is likely that the Marik Protocol will reduce the incidence and severity of this syndrome. While the cost savings are difficult to quantitate, I think that this proposition should be mentioned in the discussion section.

I have read this submission. I believe that I have an appropriate level of expertise to confirm that it is of an acceptable scientific standard.
